# Effect of Cu/Li Ratio on Mechanical Properties and Corrosion Behavior of Sc-Containing Al-Cu-Li Alloys

**DOI:** 10.3390/ma18102254

**Published:** 2025-05-13

**Authors:** Changlin Li, Xiwu Li, Yongan Zhang, Kai Wen, Lizhen Yan, Ying Li, Yanan Li, Mingyang Yu, Guanjun Gao, Hongwei Yan, Zhihui Li, Baiqing Xiong

**Affiliations:** 1 State Key Laboratory of Nonferrous Structural Materials, China GRINM Group Co., Ltd., Beijing 100088, China; lichanglin97@163.com (C.L.); wenkai@grinm.com (K.W.); yanlizhen@grinm.com (L.Y.); liying@grinm.com (Y.L.); liyanan@grinm.com (Y.L.); yumingyang@grinm.com (M.Y.); gaoguanjun@grinm.com (G.G.); yanhongwei@grinm.com (H.Y.); lizhihui@grinm.com (Z.L.); xiongbq@grinm.com (B.X.); 2 GRIMAT Engineering Institute Co., Ltd., Beijing 101407, China; 3 General Research Institute for Nonferrous Metals, Beijing 100088, China

**Keywords:** Al-Cu-Li alloy, Sc element, microstructure, strengthening mechanism, corrosion resistance

## Abstract

In this work, the effects of the Cu/Li ratio on the mechanical properties and corrosion behavior of Sc-containing Al-Cu-Li alloys were systematically investigated by utilizing age-hardening behavior, tensile property, corrosion behavior, and electrochemical behavior, complemented by microstructural characterization through EBSD and TEM. The results show that the peak aging strength of the alloys remained relatively consistent but slightly decreased with the decrease in Cu/Li ratio, and the yield strengths were 585 MPa, 578 MPa, and 573 MPa, respectively. The changes in the Cu/Li ratio caused different matching patterns of precipitates in the peak aging alloys. The cumulative precipitation strengthening by T_1_, θ′, δ′, and S′ phases are equal within the alloys with different Cu/Li ratios. However, the strength contribution of the T_1_ phase decreases from 81% to 66% with the decrease in the Cu/Li ratio. Concurrently, the precipitates of LAGBs gradually increase in number and are continuously distributed, and the precipitates of HAGBs become larger in size with lower Cu content as the Cu/Li ratio decreases, all of which leads to a weakening of the intergranular corrosion (IGC) resistance within the low Cu/Li ratio alloy.

## 1. Introduction

Al-Li alloys have gained wide attention in the aerospace industry due to their excellent ability to achieve good weight reduction without loss of strength [1,2,3]. In particular, the third generation of Al-Cu-Li alloys has been developed and applied, including 2195, 2050, and 2060, etc., which have considerable comprehensive mechanical properties, including high specific strength, high toughness, and damage tolerance, compared with the conventional 2xxx and 7xxx alloys [4,5,6,7,8]. Currently, the concentration and ratio of the main alloying elements Cu and Li and the micro-alloying elements (Mg, Ag, Sc, Zr, etc.) are mainly controlled to adjust the grain structure and precipitate characteristics to meet the performance requirements [9,10,11].

Cu and Li are the main strengthening elements of Al-Li alloys, and both of them have high solid solubility in the Al matrix at high temperatures and thus can have an excellent solid solution strengthening effect [12]. In addition, the addition of Cu and Li in aluminum alloys can generate the T_1_ phase (Al_2_CuLi), θ′ phase (Al_2_Cu), δ′ phase (Al_3_Li), and other strengthening phases [13,14], and the change in the Cu/Li ratio can have an impact on the distribution and volume fractions of intragranular and grain-boundary precipitates, which affect the strength and corrosion resistance of Al-Li alloy [15,16]. This is based on previous investigations [17,18], which summarized the precipitation sequences of different Cu/Li ratios in ternary Al-Cu-Li alloys as follows: Cu/Li ratio > 4: α(SS) → GP zones → θ″ → θ′; Cu/Li ratio = 2.5~4: α(SS) → GP zones → GP zones + δ′ → θ″ + θ′ + δ′ → δ′ + T_1;_ Cu/Li ratio = 1~2.5: α(SS) → GP zones + δ′ → θ′ + δ′ → δ′ + T_1_ → T_1_; Cu/Li ratio < 1: α(SS) → δ′ + T_1_ → T_1_. The addition of other alloying elements can also affect the precipitation process, causing it to become complex and unstable [19]. Sc is considered to be the best enhancer in Al alloys, and Sc and Zr mixed additions will also form a core-shell structure of the Al_3_(Sc, Zr) particles to strengthen the alloy [20,21,22]. Al_3_Sc/Al_3_(Sc, Zr) can play a role in refining grains and pinning grain boundaries to inhibit recrystallization in the process of deformation and heat treatment, thus contributing to improve the strength, plasticity, and corrosion resistance of alloys [23,24,25,26]. Therefore, the addition of Sc to Al-Cu-Li alloys has attracted the interests of researchers to develop alloys with good overall performance.

The T_1_ phase, as the most prominent strengthening phase of third-generation commercial Al-Li alloys, has a hexagonal lattice with parameters a = 0.496 nm and c = 0.935 nm [27]. Noble et al. [28] identified them as platelet-shaped precipitates with a habit plane of {111}_Al_. For the precipitation studies of T_1_, most of them consider that it nucleates at the dislocation; Gao et al. [29] also found that T_1_ precipitates nucleate directly from their own GPT_1_ zones and grow thicker in two possible paths by using calculations and experiments in the Al-4.15Cu-1.15Li alloy. One is regular by repeating T_1_ cells, and the other is abnormal via T_1_ variants. The θ′ phase is a semi-coherent metastable phase having a tetragonal structure with parameters a = 0.404 nm and c = 0.580 nm, which forms as octagonal plates on the {100}Al plane. Due to the presence of both Cu elements, there will be competitive precipitation of θ′ with the T_1_ phase during the aging process [30]. The δ′ phase belongs to an L1_2_ superlattice structure with a lattice constant of a = 0.4308 nm. The δ′ phase is completely coherent and has a minor mismatch with the Al matrix [31]. In general, the spherical δ′ phase precipitates uniformly in the Al matrix. When the GP zone and θ′ phase exist in the Al matrix, the δ′ phase will nucleate and grow on both sides of the GP zone and θ′ phase, forming a δ′/GP zone/δ′ or δ′/θ′/δ′ composite phase [32,33].

The strengthening mechanisms of precipitates and the associated precipitate-dislocation interactions have been reported in many studies in the literature. Early studies suggested that T_1_ and θ′ phases were similar as shear-resistant particles [34]. Dorin [35,36] found that the transition between shearing and bypassing of the T_1_ phase occurs during the over-aging condition, and this transition is related to the coarsening of the T_1_ phase, especially the increase in the T_1_ plate thickness. Zeng [37] identified that the strengthening transition from bypassing to shearing occurs in T_1_ precipitates with a thickness < 1.9 nm or diameter < 65 nm and successfully predicted the strengthening contribution of the T_1_ phase in recent research. The major mechanisms accounting for the strengthening effect of the δ′ phase are modulus mismatch strengthening and ordering hardening [38,39]. Krug et al. [40] concluded that the strength of the alloy depends mainly on these two contributions in the case of particles sheared by dislocations.

However, a coarse and harmful W(AlCuSc) phase was found in Sc-containing alloys with Cu content of 3–6 wt.%, which is a (Cu, Sc)-rich insoluble phase that consumes the Cu elements required for the formation of strengthening phases, such as the T1 and θ′ phases, and thus reduces the properties of the alloys. Previous studies [22] have found the presence of the W phase in as-cast Al-Cu-Li alloys with low Sc content (0.12 wt.%), and most of the W phase was re-dissolved into the matrix after a high-temperature homogenization heat treatment, but still a small amount of spherical W phase persisted. It can be summarized from above literature that the former research concept of reducing the generation of the W phase by heat treatment or deformation [14,20] cannot entirely inhibit the W phase. In the latter case, a good effect has been achieved by controlling the alloy composition to suppress the generation of the W phase, especially by adjusting the Cu element that constitutes the W phase. However, the variation of Cu content can also bring a change in properties of alloys due to a modification of the distribution and number density of the strengthening phases. In addition, the variation of Li can significantly affect not only the effect of Sc elements but also the type of strengthening phases. Nevertheless, the influence of the main alloying elements Cu and Li on the governing of the W phase as well as the microstructure and properties in Al-Cu-Li alloys is still insufficient, so we expect to investigate the influence of Cu/Li ratio on the microstructure and mechanical properties and corrosion properties of alloys in Sc-containing Al-Cu-Li alloys.

In this work, the tensile properties and corrosion behavior of peak aging alloys after extrusion with different Cu/Li ratios were tested and explored by controlling the total amount of Cu + Li constant in Sc-containing Al-Cu-Li alloys. A comprehensive analysis explains the influence of Cu/Li ratios on the strength and corrosion resistance of the alloys through the matching pattern of the precipitates.

## 2. Materials and Methods

**Figure 1:**
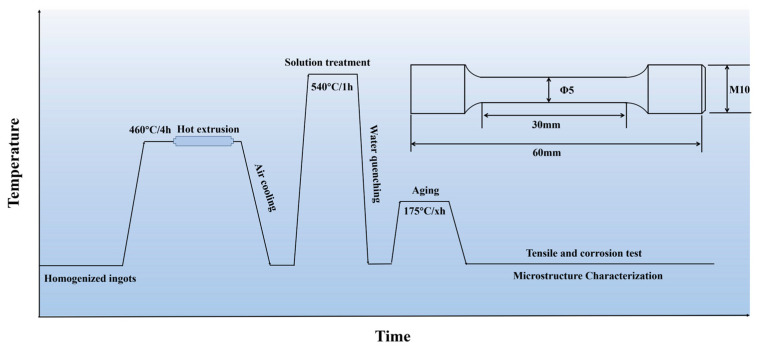
Schematic diagram of the experimental route.

Figure 1 shows the schematic diagram of the experimental route. The ingots of Al-Cu-Li alloy used for hot extrusion in the present work were prepared by melting and casting methods. Commercially pure Al, Cu, Li, Mg, Ag, and master alloys of Al-2%Sc, Al-5%Zr, and Al-10%Mn were prepared as raw materials. The raw material was put into the graphite crucible and heated to 750 °C by an electronic resistance furnace under the protection of argon gas during the whole process and then stirred sufficiently when melted completely, after which the melt was poured into the water-cooling copper mold. After cooling to room temperature, the ingots were tested for chemical composition by inductively coupled plasma-atomic emission spectroscopy (ICP-AES, Waltham, MA, USA), and the actual compositions are displayed in Table 1. Afterwards, the ingots were applied a three-stage homogenization heat treatment (300 °C/24 h + 505 °C/24 h + 530 °C/24 h) to eliminate dendritic segregation and promote a uniform distribution of the elements. The first stage was selected to promote the precipitation of fine Al_3_(Sc, Zr) dispersoids, the second stage was designed to dissolve the Cu-containing second phase, and the third stage was designed to dissolve the W phase. The homogenized ingots were pre-heated at 460 °C for 4 h before hot extrusion. The 110 mm diameter ingots were subsequently extruded into 63 mm × 16 mm plates with an extrusion ratio of 9.4:1 and an extrusion speed of 0.5 mm/s. Solid solution treatment was performed on extruded samples at 540 °C for 1 h, followed by water quenching and artificial aging at 175 °C.

Vickers hardness was tested on a WILSON VH1150 hardness tester with a load of 9.8 N and a holding time of 15 s. Tensile tests were performed on a MTS CMC 4304 universal testing machine at a tensile rate of 2 mm/min. The intergranular corrosion (IGC) test was performed according to GB/T 7998-2023 [41]. The samples were etched into a 57 g/L NaCl + 10 mL/L H_2_O_2_ (30%) solution for 6 h, and the temperature was maintained at 30 °C using a water bath. The dynamic potential polarization curves were tested using a Versa STAT 3F (Berwyn, IL, USA) electrochemical workstation, and the samples were ground and then cleaned with alcohol to remove the surface oxide layer. A saturated calomel electrode was used as the reference electrode, and platinum was used as the counter electrode. The samples were immersed in the test solution (3.5%NaCl) for half an hour before the tests and then started at a scanning rate of 1 mV/s.

Microstructure observations for electron back-scatter diffraction (EBSD) were performed on a JEOL JSM 7900F (Tokyo, Japan) field emission gun scanning electron microscope operating at 20 kV and its matching energy spectrum analysis module. The precipitates were characterized by transmission electron microscopy (TEM) in a FEI Tecnai G2 F20 (Hillsboro, OR, USA) field emission transmission electron microscope operated at 200 kV. The thickness of the observation zone in TEM specimens was measured by convergent beam electron diffraction (CBED) in order to determine the number densities and volume fractions of precipitates. Samples for TEM characterization were first mechanically polished to a thickness of about 50 μm and then prepared at −30 °C in a mixture of 30% nitric acid and 70% methanol using twin-jet electron polishing.

## 3. Results

### 3.1. Age-Hardening Behavior and Mechanical Properties

**Figure 2:**
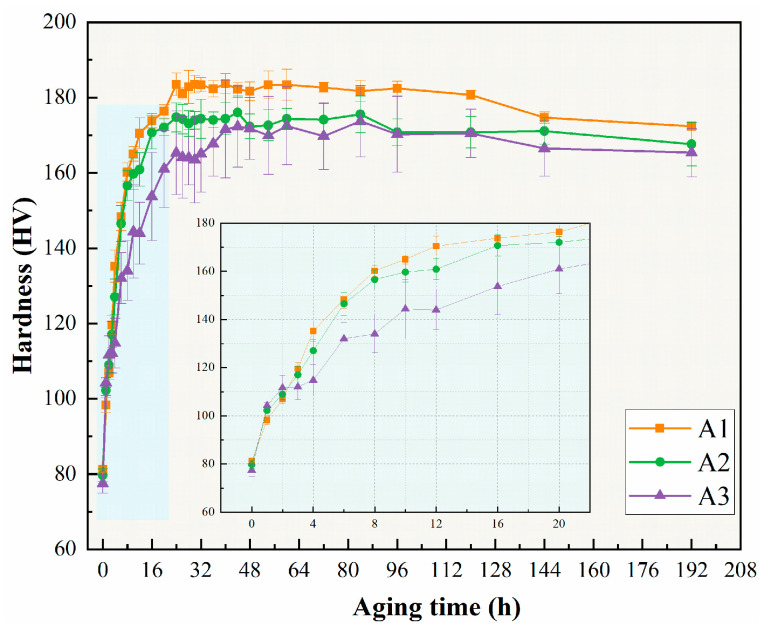
Age-hardening curves of alloys at 175 °C.

The hardness changes of three alloys under artificial aging at 175 °C are presented in Figure 2, where the first values (0 h) represent the hardness values in an as-quenched state, which are 81 HV, 80 HV, and 78 HV for the A1, A2, and A3 alloys, respectively. However, the aging response rates of the three alloys were not identical, with the A3 alloy showing the fastest increase in hardness in the early stages of aging (before 2 h). Then this trend reverses, with the A1 and A2 alloys reaching peak hardness at a relatively close aging time of 24 h. The A3 alloy shows a slower aging response and requires 60 h to obtain peak hardness. The peak hardness of the three alloys exhibits a gradual decrease with decreasing Cu/Li ratio, and the peak hardness values are 184 HV, 175 HV, and 172 HV, respectively. The hardness of all alloys reaches a peak, stabilizes for a period of time, and then slightly decreases as the aging time increases.

**Figure 3:**
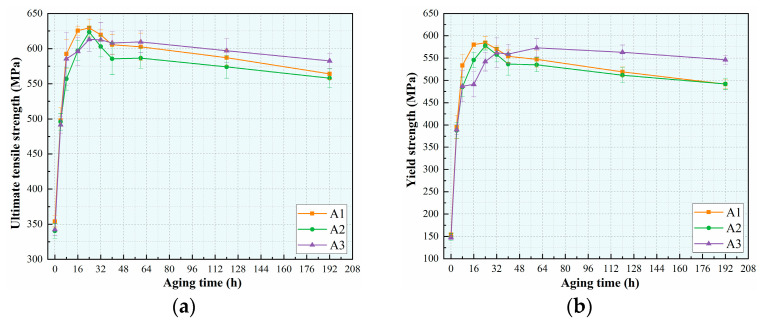

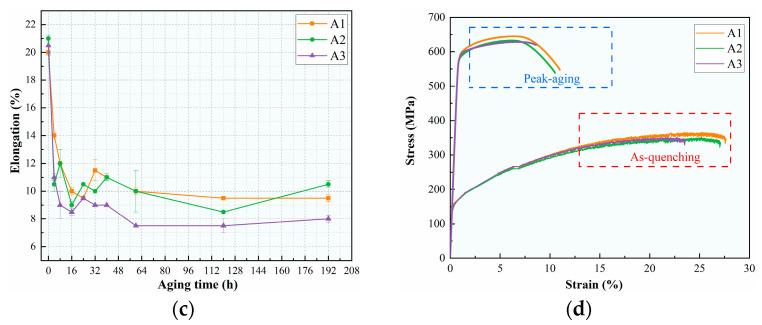
Mechanical properties of alloys at 175 °C: (**a**) Ultimate tensile strength; (**b**) yield strength; (**c**) elongation; (**d**) stress-strain curve.

Figure 3a–c show the tensile properties evolution of the three alloys during artificial aging at 175 °C. The strength trends of the A1 and A2 alloys are close to each other, and the peak strength is reached by the aging time at 24 h. The ultimate tensile strengths (UTS) of two alloys are 630 MPa and 624 MPa, the yield strengths (YS) are 585 MPa and 578 MPa, and the elongation percentages are 9.5% and 10.5%, respectively. The strength of the A3 alloy is higher than that of the A1 and A2 alloys after 40 h of aging time. The UTS, YS, and elongation of peak aging of the A3 alloy are 618 MPa, 573 MPa, and 7.5%, respectively. However, the overall elongation of the A3 alloy is lower than that of the other two alloys. Figure 3d presents the stress-strain curves of the alloys in the as-quenched and peak-aged states, while Table 2 summarizes their specific tensile properties. It can be seen that the peak aging strength values of three alloys are basically close to each other, and the yield strength increments are also consistent, which are 431 MPa, 429 MPa, and 426 MPa, respectively. Notably, the plasticity of the alloys decreases significantly with a reduction in the Cu/Li ratio.

### 3.2. Intergranular Corrosion and Electrochemical Testing

**Figure 4:**
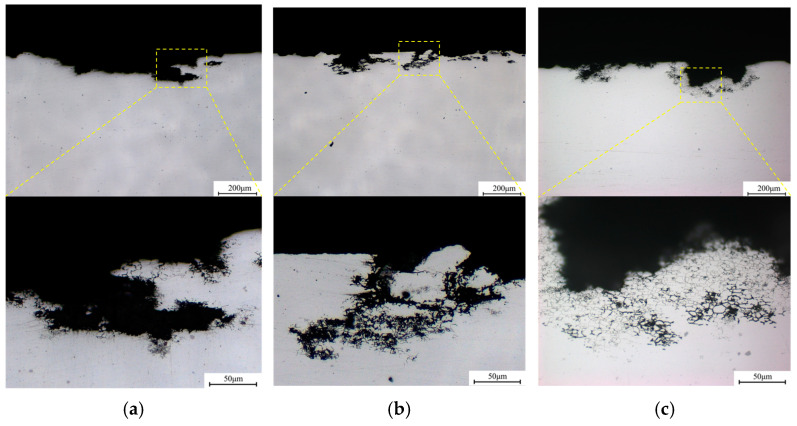
Metallographic images of typical corrosion morphology of three alloys (taken from the cross-section plane): (**a**) A1; (**b**) A2; (**c**) A3.

**Figure 5:**
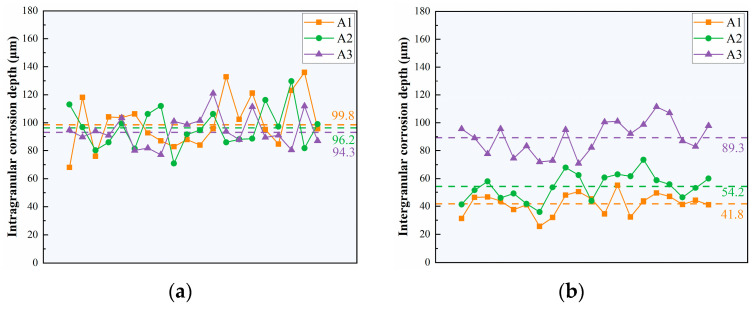
Corrosion depth statistical results: (**a**) intragranular corrosion; (**b**) intergranular corrosion.

Figure 4 shows the metallographic images of typical corrosion morphology of three alloys taken from the cross-section plane. Two corrosion modes have been discovered: intragranular corrosion and intergranular corrosion (IGC). Intragranular corrosion pits are evident in all three alloys with slight differences, but the IGC depth increases significantly as the Cu/Li ratio decreases. Figure 5 shows the results of the average corrosion depth after counting numerous metallographic images, from which it can be visualized that the intragranular corrosion average depths of three alloys show a slight decreasing trend, which are 99.8 μm, 96.2 μm, and 94.3 μm, respectively, and the IGC average depths show an elevating trend with the decrease in the Cu/Li ratio, which are 41.8 μm, 54.2 μm, and 89.3 μm, respectively.

**Figure 6:**
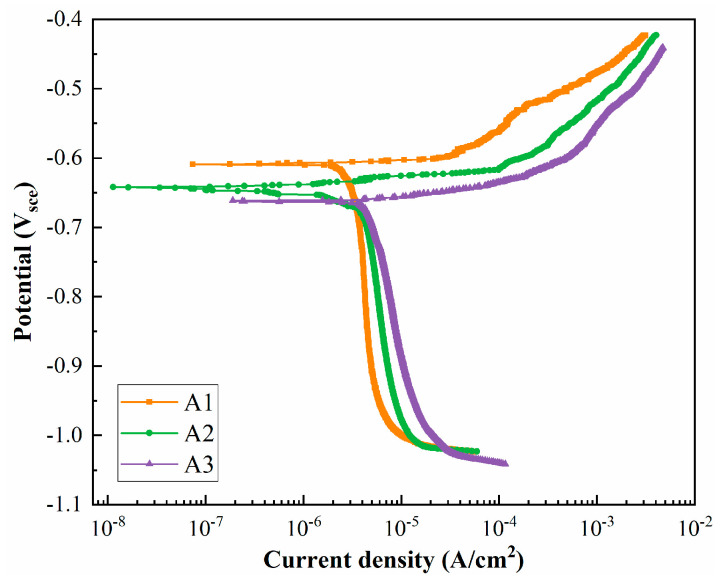
Electrochemical polarization curves of peak aging alloys.

Figure 6 shows the electrochemical polarization curves of peak aging alloys. The self-corrosion potentials (Ecorr) and self-corrosion current densities (Icorr) of different samples were obtained by extrapolating Tafel curves and are listed in Table 3. Ecorr characterizes the difficulty of the material corrosion; the more negative value indicates that corrosion occurs more easily. Icorr is used to evaluate the corrosion rate, with larger values indicating a more rapid corrosion. The Ecorr values decrease from −0.609 V to −0.662 V, and the Icorr values increase from 2.52 × 10^−5^ A/cm^2^ to 4.03 × 10^−5^ A/cm^2^, indicating that the corrosion resistance of peak aging alloys gradually weakens with the decrease in the Cu/Li ratio. These electrochemical test results are also consistent with the intergranular corrosion test results above.

### 3.3. Characteristics of Intragranular Precipitates

**Figure 7:**
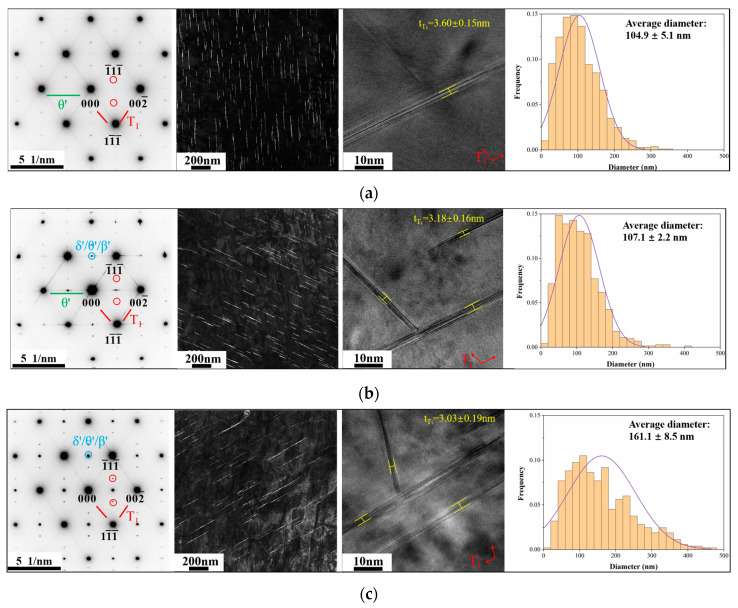
Selected areas electron diffraction (SAED) patterns of peak aging alloys along the <110>_Al_ zone axis, dark field (DF) images, HRTEM images, and size distribution statistics of T_1_ phases: (**a**) A1; (**b**) A2; (**c**) A3.

Figure 7 shows TEM images along the <110>_Al_ zone axis and T_1_ phase size distribution statistics of peak aging alloys. The lines forming the rhombuses and the spots at the 1/3 and 2/3 positions of the long diagonals of the rhombuses are found within the selected areas’ electron diffraction (SAED) patterns of all three alloys, which are the diffraction pattern characteristics of the T_1_ phase. However, there are significant differences in the number and size of the T_1_ phases as the dominant strengthening phase from dark field (DF) and HRTEM images. Table 4 lists the size and number statistics of the T_1_ phase in peak aging alloys. The average diameter and average thickness of the T_1_ phase for the A1 and A2 alloys are essentially equal, and both are significantly smaller than for the A3 alloy. However, the number density gradually decreases with the reduction in the Cu/Li ratio. The volume fractions of the T_1_ phase for the three alloys are calculated to be 2.25%, 2.13%, and 3.17%, respectively. The volume fraction (f*_v_*) of the disk-like T_1_ phase was calculated according to Equation (1) [20]:

(1) $$f_{v}=\frac{N\pi d_{T_{1}}^{2}t_{T_{1}}}{4}$$

where N is the number of the T_1_ phase per unit volume, and *d_T_*_1_ and *t_T_*_1_ are the average diameter and thickness of the T_1_ phase, respectively.

**Figure 8:**
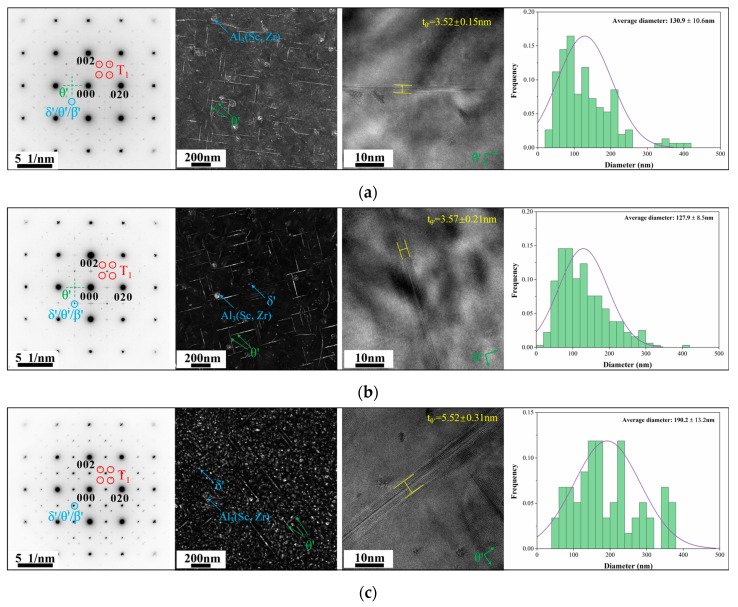
SAED patterns of peak aging alloys along the <100>_Al_ zone axis, DF images, HRTEM images, and size distribution statistics of θ′ phases: (**a**) A1; (**b**) A2; (**c**) A3.

**Figure 9:**
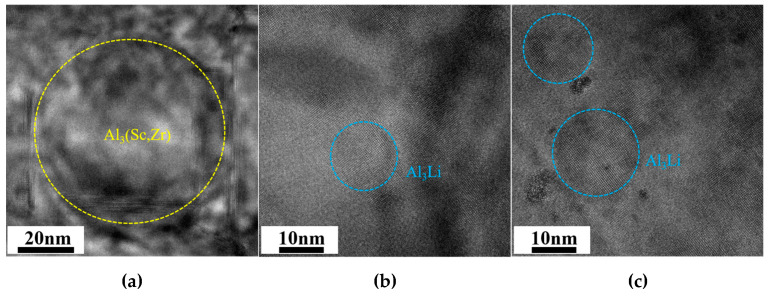
HRTEM images of Al_3_(Sc, Zr)and δ′ phases along the <100>_Al_ zone axis: (**a**) A1; (**b**) A2; (**c**) A3.

Figure 8 shows SAED patterns, DF, and HRTEM images of θ′ phases along the <100>_Al_ zone axis of peak aging alloys, as well as the statistical distribution of sizes. There are more kinds of phases within the alloys from the SAED patterns, the A2 alloy contains the highest number of the mutually orthogonal θ′ (Al_2_Cu) phase, and the A3 alloy contains the lowest number of the θ′ phase combined with the DF images. The thickness of the θ′ phase is larger within A3 alloys due to the longer time it takes for A3 alloys to reach peak aging. The Al_3_(Sc, Zr) phase with a core-shell structure is found in all three Sc-containing alloys. The δ′ (Al_3_Li) phase has also been detected in the A2 and A3 alloys, with a lower number in the A2 alloy and the highest number of the δ′ phase in the A3 alloy due to the higher Li content of the A3 alloy. Figure 9 shows the HRTEM images of Al_3_(Sc, Zr) and δ′ phases, which reveal the presence of nucleation and the growth of the θ′ phase surrounding the Al_3_(Sc, Zr) phase. The size of the δ′ phase of the A2 alloy is lower than that of the A3 alloy, as seen by comparing the HRTEM images. Statistical results on the size and number density of θ′ and δ′ phases in peak aging alloys are presented in Table 5. The number density of the θ′ phase exhibits a tendency of first increasing and then decreasing as Cu/Li decreases, and the volume fractions of the θ′ phases of the three alloys are 0.89%, 1.08%, and 0.50%, respectively. The δ′ phase is found only in the A2 and A3 alloys, where the volume fractions are 0.07% and 1.46%.

**Figure 10:**
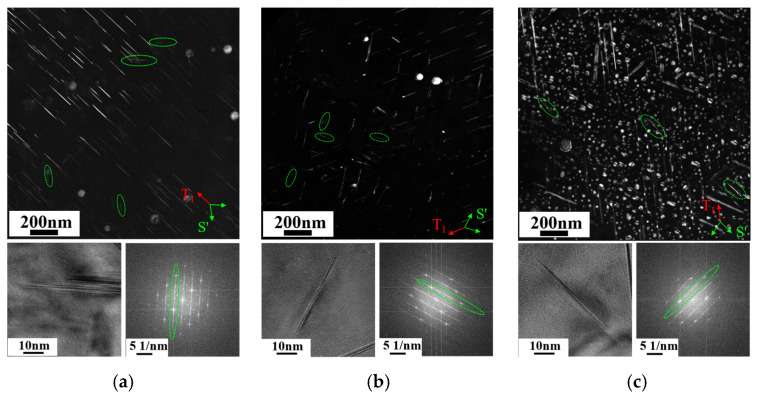
DF images and HRTEM images and corresponding SAED patterns of S′ phase along <112>_Al_ zone axis: (**a**) A1; (**b**) A2; (**c**) A3.

As the alloys contain about 0.4 wt.% of Mg, the presence of the S′ (Al_2_CuMg) phase is also found within the Al-Cu-Li alloys, which has an orthorhombic structure and forms on {012}_Al_ habit planes and grows along the <100>_Al_ directions. The S′ phase exhibits a rod-like shape with 12 variants within Al alloys. In order to easily distinguish it from other phases, the TEM observations were carried out along the <112>_Al_ zone axis to characterize the S′ phase. It can be seen from Figure 10a–c that S′ phases are present in two directions within all three alloys, which are fewer in number and smaller in size, and the overall morphology of the alloy shows a small variance owing to the consistent content of Mg. The HRTEM images and SAED patterns both demonstrate that they are two variants of S′ along the <042>_Al_ direction. The S′ phase diameters of all three alloys were counted to be about 51 ± 2.2 nm.

### 3.4. Characterization of Grain Boundary Precipitates

**Figure 11:**
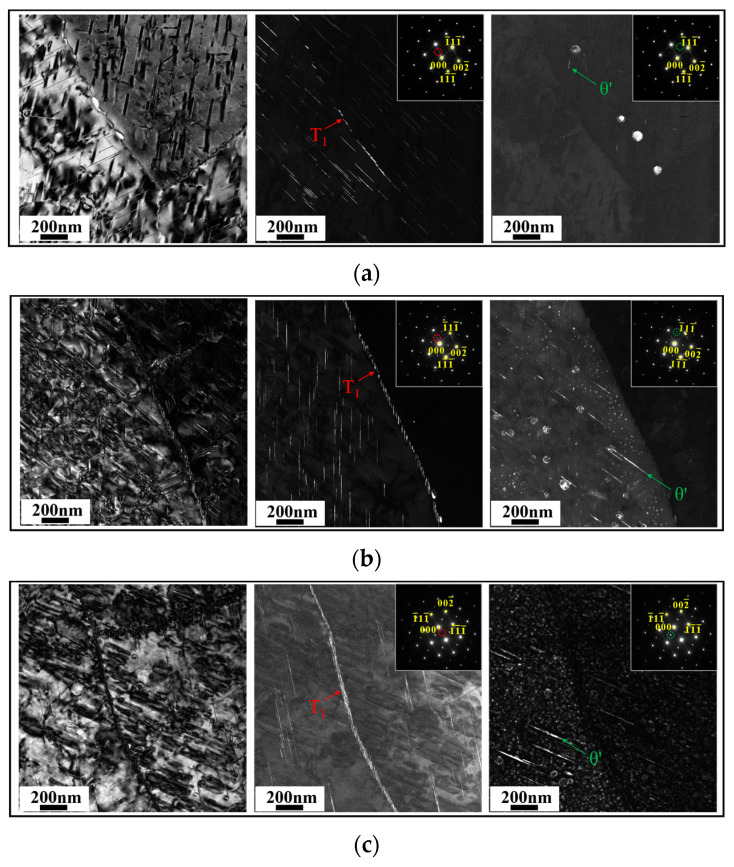
BF and DF images from around the LAGBs along the <110>_Al_ zone axis: (**a**) A1; (**b**) A2; (**c**) A3.

**Figure 12:**
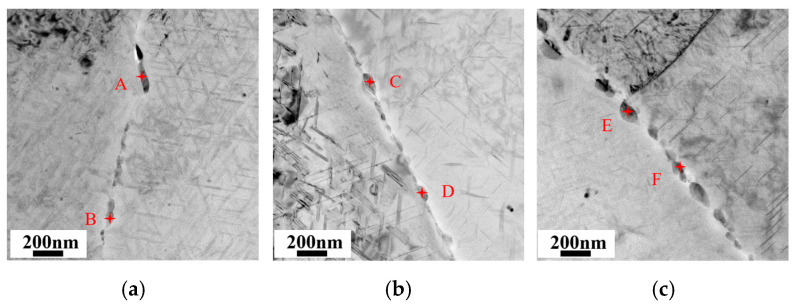
BF images from around the HAGBs: (**a**) A1; (**b**) A2; (**c**) A3.

In order to further investigate the effect of the Cu/Li ratio on grain boundary precipitates (GBPs), TEM observation experiments were carried out on the sub-grain boundaries (SGBs) and grain boundaries (GBs) of all three alloys. Figure 11 demonstrates the precipitate morphology of SGBs along the <110>_Al_ zone axis. From the DF images, it can be found that the precipitates of SGBs are mainly the T_1_ phase, which significantly increases in number and gradually decreases in size, and the distribution is continuous with the decrease in the Cu/Li ratio. No obvious enrichment of θ′ and δ′ has been detected on the SGBs. Figure 12 shows the BF images from the vicinity of the GBs, and Table 6 lists the EDS results for the precipitates at the marked locations. The difference in the size of GBPs between the A1 and A2 alloys is relatively slight, but the A2 alloy exhibits a higher numerical density of them. The A3 alloy has the highest numerical density of precipitates, and their size is larger on the GBs. It can be concluded from the EDS results that the precipitates on the GBs are mainly Al2Cu phases and AlCu phases containing Mg and Ag. The Li element is not fully analyzed due to its low atomic order. However, the contents of Cu elements in the GBPs also decrease overall with the decrease in the Cu/Li ratio, which also affects the corrosion resistance of the alloy, and this will be discussed subsequently.

## 4. Discussion

### 4.1. Effect of Cu/Li Ratio on Microstructure

**Figure 13:**
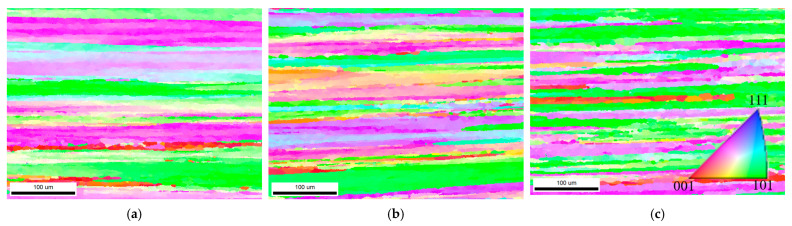

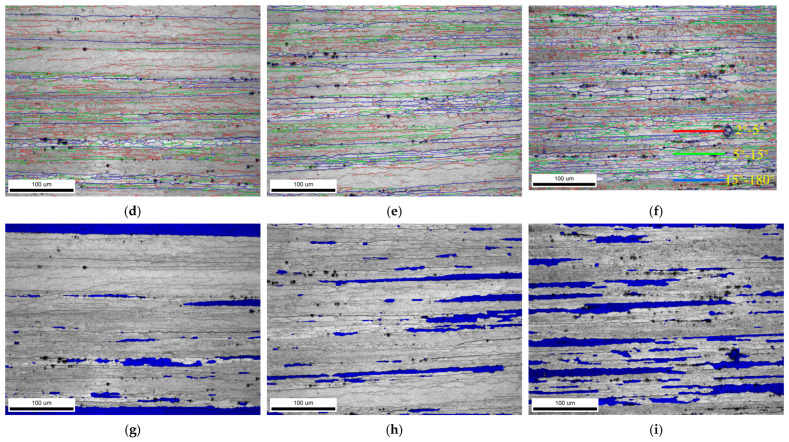
EBSD images of the solid solution and aging-treated extruded Al-Cu-Li alloys: (**a**,**d**,**g**) A1; (**b**,**e**,**h**) A2; (**c**,**f**,**i**) A3; (**a**–**c**) inverse pole figure [001]; (**d**–**f**) boundaries: rotation angle; (**g**–**i**) grain orientation spread.

Extensive studies have shown that the addition of Sc, Zr elements within Al alloys can cause grain refinement, which is attributed to the fact that primary Al_3_(Sc, Zr) particles formed during solidification can act as heterogeneous nucleation sites [21,42,43,44]. Figure 13 shows the EBSD images of extruded Sc-containing Al-Cu-Li alloy after solid solution and aging treatment along the extrusion direction. From the inverse pole figure (IPF) of three alloys, it can be seen that the alloy grains exhibit a fibrous structure aligned along the extrusion direction. While the difference in grain thickness between the A1 and A2 alloys is negligible, with both displaying similar grain characteristics, the grain size of the A3 alloy (with a low Cu/Li ratio) is significantly reduced. Radmilovic et al. [45]. highlighted that the addition of Li increases the driving force for Al_3_Sc nucleation. This is attributed to Li reducing the solid solubility of Sc in the α-Al matrix, leading to higher supersaturation of Sc. In addition, the decrease in the Cu element also affects the solid solubility of the Sc element in the Al matrix. Although the total weight percentage of Cu and Li elements was maintained constant in this study, the substantial increase in Li atomic percentage accompanying the decreased Cu/Li ratio (particularly in A3 alloys) reduces the solid solubility of Sc in the matrix. This promotes precipitation of primary Al_3_(Sc, Zr) particles, consequently enhancing grain refinement. Figure 13d–f presents the grain boundary reconstruction maps for the three alloys, where boundaries with misorientation angles between 15° and 180° are identified as high-angle grain boundaries (HAGBs). The relative fraction of these HAGBs serves as an indicator of the alloys’ recrystallization degree. Correspondingly, Figure 13g–i displays the grain orientation spread (GOS) maps, where the blue grains (0–2°) represent the recrystallized grains. Table 7 shows the EBSD-derived grain boundary angle statistics and average grain orientation statistics. Combined with the corresponding pictures in Figure 13, the A1 and A2 alloys demonstrate similar distributions of high-angle grain boundaries (>15°) to low-angle grain boundaries (2–15°) and comparable recrystallization degrees. In contrast, the A3 alloy exhibits both a significantly higher HAGB fraction and greater recrystallization extent, indicating that partial dynamic recrystallization occurred during thermo-mechanical processing. He et al. [46] found that the initial grain size plays a critical role in determining the dominant dynamic recrystallization (DRX) mechanism during deformation in Al-Zn-Mg-Cu alloys. Fine-grained alloys generally exhibit a higher tendency for discontinuous dynamic recrystallization (DDRX) due to their abundant grain boundaries, which facilitate strain localization and nucleation of new defect-free grains. In this study, the A3 alloy demonstrates the finest initial grain structure and exhibits the highest degree of recrystallization following extrusion and solid solution treatment. In addition, the variation in grain size causes differences in the number of grain boundaries. The finer initial grain size of the A3 alloy promotes more pronounced dislocation pile-up near grain boundaries during deformation, increasing the LAGB density, which also affects the corrosion resistance of Sc-containing Al-Cu-Li alloys [15,47].

The differences in aging precipitates were explored in this study by controlling the total amount of Cu + Li consistently and changing the Cu/Li ratios. The variation in Cu/Li ratios causes the precipitates of peak aging alloys to have different types, numbers, and size matching. Due to the large element types within the alloys, the precipitates have a wide range of types including T_1_, θ′, δ′, and S′ phases. It has been shown in previous studies that a high Cu/Li ratio contributes to the precipitation of the T_1_ phase [15]. Although the T_1_ phase is the dominant strengthening phase in all three alloys, as seen in Figure 9, the T_1_ phase number density decreases and the average diameter increases as Cu/Li decreases in the alloys. Because of the competitive relationship between T_1_ and θ′ phases during aging, the high number density of the T_1_ phase inhibits θ′ generation, and thus the θ′ phase within the A1 alloy with a high Cu/Li ratio is lower than that of the A2 alloy. The δ′ phase was also found within the A2 alloy due to the increasing content of Li. As the Cu content is further reduced and the Li content is further increased, the main precipitates within the A3 alloy transform into a large number of δ′ and T_1_ phases and minor amounts of the θ′ phase. Meanwhile, minor amounts of the S′ phase were observed in all alloys. The number densities of Al_3_(Sc, Zr) phases were not explored in detail due to the relatively low elemental content of Sc and Zr that are common to the alloys, resulting in relatively small numbers of Al_3_(Sc, Zr) phases with minor variations in each alloy. The changes in the Cu/Li ratio altered the matching pattern of the strengthening precipitates, thus affecting the properties of peak aging alloys.

### 4.2. Effect of Cu/Li Ratio on Mechanical Properties

Based on the above results, the peak aging strengths of the three alloys tend to be approximately similar but slightly decreasing in spite of the changes in Cu/Li that lead to changes in the grains and precipitates of the alloys, and this strengthening mechanism is attributed to grain refinement strengthening, solid solution strengthening, and precipitation strengthening. The current yield strength of the Al-Cu-Li alloys for each strengthening contribution mechanism is expressed as [14,48,49]:

(2) $$\sigma_{YS}=\sigma_{b}+\sigma_{\mathrm{ps}}$$

(3) $$\sigma_{\mathrm{ps}}=\sigma_{T1}+\sigma_{\theta^{\prime }}+\sigma_{\delta^{\prime }}+\sigma_{\;s^{\prime }}$$

where *σ_YS_* is the total yield strength, *σ*_b_ is the base strength resulting from the combined contribution of solid solution strengthening and the grain boundary strengthening, and *σ*_ps_ is the precipitation strengthening. Considering that the change in solid solution strengthening is less in the aging procedure, it is reasonable to assume that *σ*_b_ is obtained from the yield strength of the alloy at room temperature tensile after quenching. The yield strength increments obtained from Table 2 compared to peak aging alloys can be considered as precipitation strengthening contributions. The ∆_YS_ values of the three alloys are relatively close to each other; however, the differences in the precipitates are significant, so we need to quantify the strength contributions of the individual precipitated phases. Precipitation strengthening only takes into account these four dominant T_1_, θ′, δ′, and S′ phase strength contributions.

Precipitation strengthening is the dominant strengthening mode in Al-Cu-Li alloys, which is essentially the interaction of precipitates with dislocations [50,51]. The strengthening mechanism of the T_1_ phase is more complex, with early studies suggesting that the T_1_ phase is unshearable particles like the θ′ phase. Dorin et al. [36] revealed a transition from shearing to bypassing in the dislocation/T_1_ interaction, which is related to the thickness of the T_1_ phase. Zeng [37] suggested that this mechanism of transition occurs when the thickness of T_1_ precipitates is less than 1.9 nm or the diameter is less than 65 nm. Therefore, a new model was proposed to calculate the strengthening contribution of the T_1_ phase, taking into account both the shearing mechanism and the bypassing mechanism as follows [20,52,53]:

(4) $$\begin{array}{ll} \sigma_{T_{1}} & =\sigma_{shear}+\sigma_{by-pass}\\ & =k(\frac{1.211Md_{T_{1}}\gamma_{eff}^{3/2}}{t_{T_{1}}^{2}}\sqrt{\frac{bf_{T_{1}}}{\Gamma }})+(1-k)\frac{0.12MGb}{\sqrt{d_{T_{1}}t_{T_{1}}}}(\sqrt{f_{T_{1}}}+0.7\sqrt{\frac{d_{T_{1}}}{t_{T_{1}}}}f_{v}+0.12\frac{d_{T_{1}}}{t_{T_{1}}}f_{T_{1}}^{3/2})\ln(\frac{0.097d_{T_{1}}}{b}) \end{array}$$

where *γ_eff_* represents the effective interfacial energy term (~0.107 J/m^2^), Γ represents the dislocation line tension and is approximately 1/2 Gb^2^, and k is the proportion of the T_1_ phase by shearing. *M* is the average Taylor factor listed in Table 7. Other relevant parameters for the T_1_ phase are taken from Table 4. The calculated values that contribute to the T_1_ phase strength for the three alloys are 348 MPa, 314 MPa, and 280 MPa, respectively.

The contribution of θ′ relative to the yield strength can be expressed by the Orawan equation as follows [34]:

(5) $$\begin{array}{l} \sigma_{\theta^{\prime }}=\frac{K_{\theta^{\prime }}}{0.931\sqrt{\frac{0.306\pi t_{\theta^{\prime }}}{f_{\theta^{\prime }}}}\sqrt{d_{\theta^{\prime }}}-\frac{\pi }{8}{\left(\sqrt{d_{\theta^{\prime }}}\right)}^{2}-1.061t_{\theta^{\prime }}}\\ K_{\theta^{\prime }}=(\frac{Gb}{2\pi \sqrt{1-v}})(\ln\frac{1.225t_{\theta^{\prime }}}{2b}) \end{array}$$

where *υ* is Poisson’s ratio (0.339), and t*_θ_*_′_, *d_θ_*_′_, and *f_θ_*_′_ are the average thickness, diameter, and volume fraction of the θ′ precipitates, respectively. The strengthening mechanism of the δ′ phase has been controversial, and according to Zhao’s [38,54] study, dislocations become curved because of δ′ phase coarsening significantly within peak aging alloys, whose strengthening model is as follows:

(6) $$\sigma_{\delta^{\prime }}=\frac{0.95\gamma_{apb}\sqrt{f_{\delta^{\prime }}}}{(2.275-2\sqrt{f_{\delta^{\prime }}})b}$$

where *γ_apb_* is the δ′ phase antiphase boundary energy (~0.158 J/m^2^), and *f_δ_*_′_ is the volume fraction of the δ′ phase. The *σ_θ_*_′_ values obtained for the three alloys were 67 MPa, 78 MPa, and 33 MPa, respectively. The *σ_δ_*_′_ values obtained for the A2 and A3 alloys are 20 MPa and 94 MPa.

It is difficult to shear the S′ phase by the dislocation, so the Orowan model is given by Khan as follows, according to the bypass mechanism [13,55,56]:

(7) $$\sigma_{S^{\prime }}=\frac{0.112MGb}{d_{S^{\prime }}}\ln\left(\frac{1.316d_{S^{\prime }}}{r_{0}}\right)\left(f_{S^{\prime }}^{1/2}+0.94f_{S^{\prime }}+2.44f_{S^{\prime }}^{3/2}\right)$$

where *d_S_*_′_ and *f_S_*_′_ are the average diameter and volume fraction of the S′ phase, and *r*_0_ is the inner cut-off radius for calculation of the dislocation line tension (equal to the Burgers vector). Since the number and size of the S′ phases of all three alloys are close to each other, the calculated S′ phase contributions to the yield strength are about 14 MPa for each alloy.

**Figure 14:**
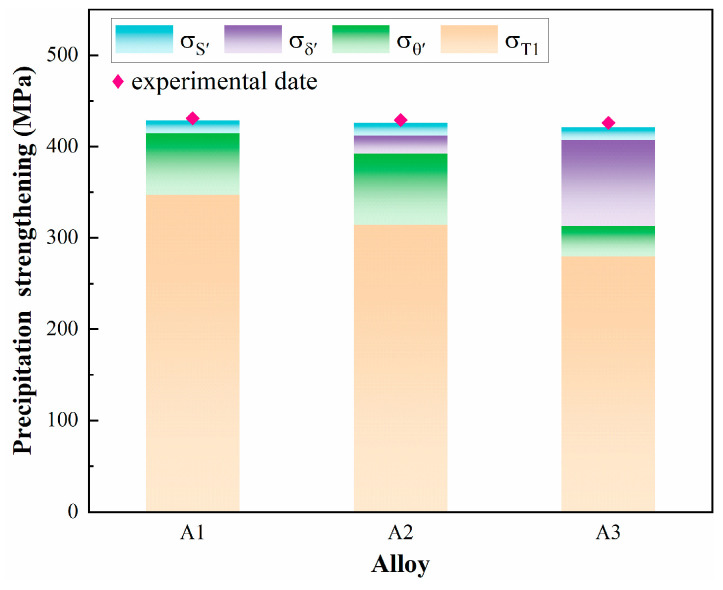
Comparison between the theoretical model of the strengthening effect and the measured ∆_YS_ values for samples with different Cu/Li ratios.

Figure 14 shows the comparison between the theoretical model of the precipitation strengthening effect and the measured ∆_YS_ values of peak aging alloys. The calculated strengths of the three alloys are 428 MPa, 426 MPa, and 421 MPa, respectively, which are extremely close to the experimental data values. Decreasing the Cu/Li ratio of alloys while controlling for a consistent total Cu + Li concentration obtained different precipitate matching patterns, and it is interesting to note that the strengths of the alloys remained at essentially the same level. Although the precipitation strengthening effect of the T_1_ phase decreases (81%, 74%, and 66%, respectively), the strength increments due to the θ′ and δ′ phases compensate for this difference with the decrease in Cu/Li ratio. However, the elongation of the A3 alloy shows a significant decrease, which is also related to the higher Li content-induced high number of δ′ phases. δ′ phase is more prone to be sheared by dislocations in the same slip plane [45,57], resulting in the inhomogeneous deformation and brittle failure of the alloys, which also explains the low plasticity of the A3 alloy.

### 4.3. Effect of Cu/Li Ratio on Corrosion Behaviors

Based on the IGC test results and electrochemical characterization, it is demonstrated that the corrosion resistance of peak aging alloys decreases with the decrease in the Cu/Li ratio, and these differences can be attributed to the grain characteristics and intragranular and grain boundary precipitates related to the alloys.

**Figure 15:**
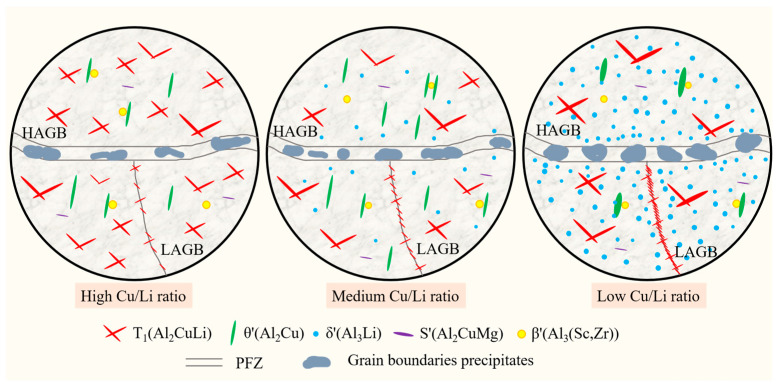
Schematic diagrams of intragranular and grain boundary precipitates characteristics of alloys with different Cu/Li ratios.

The main corrosion mechanism of the current Al alloys is anodic dissolution [7,58], and the potentials of the matrix, PFZ, and GBPs within the alloy are different. GBPs have a more negative potential and therefore prioritize dissolution as an anode to form a corrosion channel, and thus a large number of continuous precipitates on the grain boundaries affect the corrosion resistance of the alloy [15,16,47]. The change in the composition of the alloy also affects the variation in the GBPs composition, thus affecting the potential. Chen et al. [59,60] concluded that high Cu content of GBPs and a wide PFZ reduce the electrochemical reaction, thus inhibiting the corrosion occurrence within Al-Li alloys. In the current study, the variation in the Cu/Li ratio induced the differences in grain characteristics among Sc-containing Al-Cu-Li alloys, and A3 alloys with low Cu/Li ratios had a higher percentage of HAGBs. It is obvious from Figure 11 that the GBPs in LAGBs are mainly the T_1_ phase, which gradually increases in number and becomes continuously distributed with the decrease in Cu/Li. Meanwhile, the number and size of GBPs in HAGBs also gradually increase, and their Cu content appears to decrease significantly. Figure 15 shows a schematic diagram of intragranular and grain boundary precipitate characteristics for alloys with different Cu/Li ratios. The evolution of these precipitates in the grain and sub-grain boundaries affects the IGC characteristics of the alloys, and these precipitates preferentially form corrosion channels in the corrosive environment, thereby causing an increase in the corrosion depth of the IGCs with a decrease in the Cu/Li ratio. At the same time, the higher proportion of LAGBs within the A1 alloys makes it easier for corrosion to extend into the intragranular, which is combined with the high T_1_ phase number density within the A1 alloy. The intragranular corrosion depths of the alloys are basically similar but slightly decrease with the decrease in the Cu/Li ratio. However, the overall corrosion resistance of the three alloys shows a decrease as the Cu/Li ratio decreases by combining the intragranular and intergranular corrosion conditions, which is further confirmed by the electrochemical characteristics in Figure 6.

## 5. Conclusions

In the present work, the effects of the Cu/Li ratio on mechanical properties and corrosion behavior were studied in Sc-containing Al-Cu-Li alloys. The main conclusions are as follows:

(1) The peak aging strength of the alloys remained relatively consistent but slightly decreased with the decrease in the Cu/Li ratio. The yield strengths were 585 MPa, 578 MPa, and 573 MPa, respectively; however, the elongation of the alloys with low Cu/Li ratios appears to be significantly decreased.
(2) The changes in the Cu/Li ratio caused different matching patterns of precipitates in the peak aging alloys. The main precipitates of high Cu/Li ratio alloys are T_1_ and θ′ phases; the main precipitates of medium Cu/Li ratio alloys are T_1_, θ′ phases, and a small amount of δ′ phases; and the main precipitates of low Cu/Li ratio alloys are δ′ phases, T_1_ phases, and a small amount of θ′ phases. Meanwhile, minor amounts of the S′ phase are observed in all alloys.
(3) The cumulative precipitation strengthening by T_1_, θ′, δ′, and S′ phases is equal within the alloys with different Cu/Li ratios, which are 428 MPa, 426 MPa, and 421 MPa, respectively; however, the strength contribution of the T_1_ phase decreases from 81% to 66% with the decrease in the Cu/Li ratio.
(4) As the Cu/Li ratio decreases, the precipitates of LAGBs gradually increase in number and are continuously distributed, and the precipitates of HAGBs become larger in size with lower Cu content, all of which leads to a weakening of the IGC resistance within the low Cu/Li ratio alloy.

## Figures and Tables

**Table 1:** Chemical composition for investigated alloy (wt.%).

Alloys	Cu	Li	Sc	Mg	Ag	Zr	Mn	Al	Cu + Li	Cu/Li	Cu (at.%)	Li (at.%)
A1	3.90	1.28	0.11	0.37	0.30	0.12	0.22	Bal.	5.18	3.05	1.64	4.92
A2	3.44	1.67	0.11	0.39	0.29	0.11	0.21	Bal.	5.11	2.06	1.43	6.42
A3	3.03	2.11	0.11	0.38	0.29	0.11	0.21	Bal.	5.14	1.44	1.27	8.11

**Table 2:** Tensile properties of three alloys at as-quenching and peak-aging states.

Alloy	Condition	UTS (MPa)	YS (MPa)	EL (%)	ΔYS (MPa)
A1	As-quenching	354 ± 8	154 ± 4	20.0 ± 0	431
	Peak-aging	630 ± 12	585 ± 11	9.5 ± 0	
A2	As-quenching	341 ± 10	149 ± 8	21.0 ± 0.25	429
	Peak-aging	624 ± 8	578 ± 9	10.5 ± 0	
A3	As-quenching	343 ± 7.5	148 ± 5	20.5 ± 0.75	426
	Peak-aging	618 ± 9	573 ± 12	7.5 ± 0	

**Table 3:** Parameters of electrochemical polarization of peak aging alloy.

Alloys	Ecorr (V)	Icorr (A/cm^2^)
A1	−0.609	2.52 × 10^−5^
A2	−0.642	3.15 × 10^−5^
A3	−0.662	4.03 × 10^−5^

**Table 4:** Statistical results on the size and number density of the T_1_ phase in peak aging alloys.

Alloys	Average Diameter (nm)	Number Density (×10^21^ m^−3^)	Average Thickness (nm)	Volume Fraction (%)
A1	104.9 ± 5.1	1.73 ± 0.08	1.51 ± 0.15	2.25
A2	107.1 ± 2.2	1.52 ± 0.10	1.58 ± 0.16	2.13
A3	161.1 ± 8.5	0.67 ± 0.15	2.31 ± 0.19	3.17

**Table 5:** Statistical results on the size and number density of θ′ and δ′ phases in peak aging alloys.

Alloys	θ′ (Al_2_Cu)				δ′ (Al_3_Li)		
	Average Diameter (nm)	Number Density (×10^20^ m^−3^)	Average Thickness (nm)	Volume Fraction (%)	Average Diameter (nm)	Number Density (×10^21^ m^−3^)	Volume Fraction (%)
A1	130.9 ± 10.6	1.89 ± 0.12	3.52 ± 0.15	0.89			
A2	127.9 ± 8.5	2.37 ± 0.14	3.57 ± 0.21	1.08	14.0 ± 0.9	0.52 ± 0.06	0.07
A3	190.2 ± 13.2	0.24 ± 0.09	5.52 ± 0.31	0.50	16.1 ± 1.2	6.71 ± 0.18	1.46

**Table 6:** EDS results of precipitates from HAGBs (at.%).

Point	Al	Cu	Mg	Ag
A	65.60	34.27	0.08	0.05
B	72.83	19.34	6.62	1.21
C	78.08	17.93	3.23	0.76
D	69.09	24.04	5.78	1.09
E	85.28	11.32	3.09	0.09
F	86.99	9.90	0.78	0.29

**Table 7:** EBSD statistical results.

Alloys	LAGBs (%)	HAGBs (%)	Recrystallisation Ratio (%)	Average Taylor Factor
A1	71.8	28.2	7.2	3.31
A2	72.3	27.7	6.9	3.35
A3	55.5	44.5	15.3	3.15

## Data Availability

The raw data supporting the conclusions of this article will be made available by the authors on request.
